# Fasting and weight loss: mobile application-based approach

**DOI:** 10.1186/s40795-022-00645-1

**Published:** 2022-12-08

**Authors:** Sarunas Valinskas, Kasparas Aleknavicius, Marius Nakrys, Justinas Jonusas

**Affiliations:** 1 Kilo.Health, Antakalnio G. 17, 10312 Vilnius, LT Lithuania; 2 Lithuania Business University of Applied Sciences, Turgaus St. 21, 91249 Klaipeda, LT Lithuania

**Keywords:** Fasting, Weight loss, Mobile application, Dofasting, Obesity, Overweight

## Abstract

**Background:**

The purpose of this study was to investigate the efficacy of intermittent fasting (IF) guidance, delivered through a smartphone application, in terms of engagement and weight loss.

**Methods:**

We performed a retrospective chart-review study of all consecutive users with overweight or obesity of the DoFasting mobile application, which integrates IF solutions for people looking to lose weight. Users with overweight and obesity at the beginning of application use, who met the inclusion criteria (entered their gender, height, and starting weight; had more than one weight entry; used the app for at least a month; had at least one active day per week with valid app-related activities) and used the application for weight loss were included in the study.

**Results:**

The final study cohort comprised 22,022 consecutive users. The short-term cohort comprised 17,221 users (8585 females and 8636 males), while 4801 users (2983 females and 1818 males) were in the long-term cohort. Long-term active users of the application lost a statistically significant amount of weight over the study period and lost more than inactive users did (2.2 [3.5] vs 1.4 [3.2], *p* < 0.0001; the results are shown in kilograms as medians with the interquartile range in brackets). Active and long-term users were also more likely to reduce their BMI class (*p* < 0.05) in comparison to non-active short-term users. A multiple regression model showed that the total length of use and active days were the most significant predictors of weight loss. In-app activities such as total fasting hours, weight logging, completing challenges, and providing feedback were also statistically significant predictors of weight loss with varying contributions.

**Conclusion:**

We found that the DoFasting mobile application that implements IF is an effective tool for weight loss if used actively and sufficiently.

**Trial registration:**

This retrospective chart review study was approved by BRANY IRB in January 2022 (study ID.: 22‐08‐034‐939).

## Background

Intermittent fasting (IF) has gained considerable popularity in recent years. Although it is an old method traditionally used for religious reasons [1], it is now trending as a diet for its supposed various health benefits, mainly weight loss [2]. There are many regimes of IF, but the most studied are the 5:2 regime, alternate-day fasting (ADF) or alternate-day modified fasting (ADMF), and time-restricted eating (TRE) [3]. The 5:2 regime comprises reducing caloric intake to 0–25% on two non-consecutive fast days in a week [4]. ADF involves fasting every other day with no caloric intake [5]. ADMF modifies the ADF regime by including a short eating window to consume 20–25% of daily caloric intake during fast days [6]. TRE restricts the eating window every day to 8–12 h and, unlike the other fasting methods, less frequently imposes a caloric restriction (CR) [3, 7].

The main goal of IF is usually weight loss. IF generally leads to weight loss because the regimen often results in reduced caloric intake, similarly to CR diets [8]. Unlike IF, however, regular CR diets do not include constraints on the eating window. A review of intervention trials has found that almost any IF regimen results in significant weight loss [9]. However, it is currently unclear whether IF is superior to regular CR diets in this regard. A meta-analysis of fasting interventions for the treatment of overweight and obesity in adults showed that IF is similar to continuous CR regarding short-term weight loss [10]. However, human trials show that with similar caloric intake, IF can lead to more significant weight loss and improvements in insulin sensitivity and cholesterol than a CR diet can [11–13]. Moreover, IF was shown to be superior to regular CR in terms of longevity in animal models [14]. Finally, an experimental animal study found that TRE prevents excessive weight gain and has additional protective metabolic benefits even when caloric intake is not reduced [15]. Similar results have been observed in a human TRE trial [16].

Generally, IF trials deliver dietary interventions through written and/or verbal instructions and monitoring. However, it is conceivable that relatively simple IF management can be effectively provided through mobile applications, especially considering the ubiquity of smartphones. A randomized controlled trial has demonstrated that adherence to a dietary weight loss intervention is significantly higher when delivered through a smartphone application [17]. Additionally, it was demonstrated in a survey that nutrition app use is associated with more motivation and engagement when adhering to a diet [18]. Considering these aforementioned results, it can be hypothesized that using an IF app should also be associated with weight loss through improved diet adherence. Nonetheless, data on the adherence to and effectiveness of mobile IF guidance are scarce. To the best of our knowledge, there currently are no published studies about IF facilitated by a mobile application. We hope to add to the existing knowledge about the effectiveness of weight loss with IF and also examine if its results are replicable with mobile app interventions, which are becoming increasingly popular. Therefore, the purpose of this study was to investigate the efficacy of IF guidance delivered through a smartphone application in terms of both weight loss and engagement with the app. To this end, a retrospective analysis of user data was done.

## Methods

This retrospective study was conducted according to the guidelines of the Declaration of Helsinki and approved by BRANY IRB (study ID.: 22‐08‐034‐939) in January 2022.

The mobile application used in the investigation was DoFasting, developed by KiloHealth in cooperation with certified nutritionists and physicians. It creates integrated intermittent fasting solutions for people looking to lose weight and improve their overall health. DoFasting offers continuous support, education, and guidance on IF, nutrition, and training. The application provides a fasting timer, a healthy meal recipes gallery, different levels of workouts, educational articles, calorie, step, and weight loggers, weekly and monthly health reports, and chat access to certified nutritionists. The application intends to help its customers reach their health-related goals, including healthy weight management. Once the goal is reached, the application intends to assist in forming healthy habits that can be described as a sustainable way of living. This specific application was chosen due to the availability of data directly from the developers.

Users with overweight and obesity who started using the application between 1 January 2020 and 1 January 2021 and met the following criteria were included in the investigation: entered their gender, height, and starting weight; had more than one weight entry; used the app for at least a month; had at least one active day per week with at least one valid app-related activity (e.g., documented activities, entered data, completed exercise from the app, looked for nutrition information in the app, etc.).

**Fig. 1:**
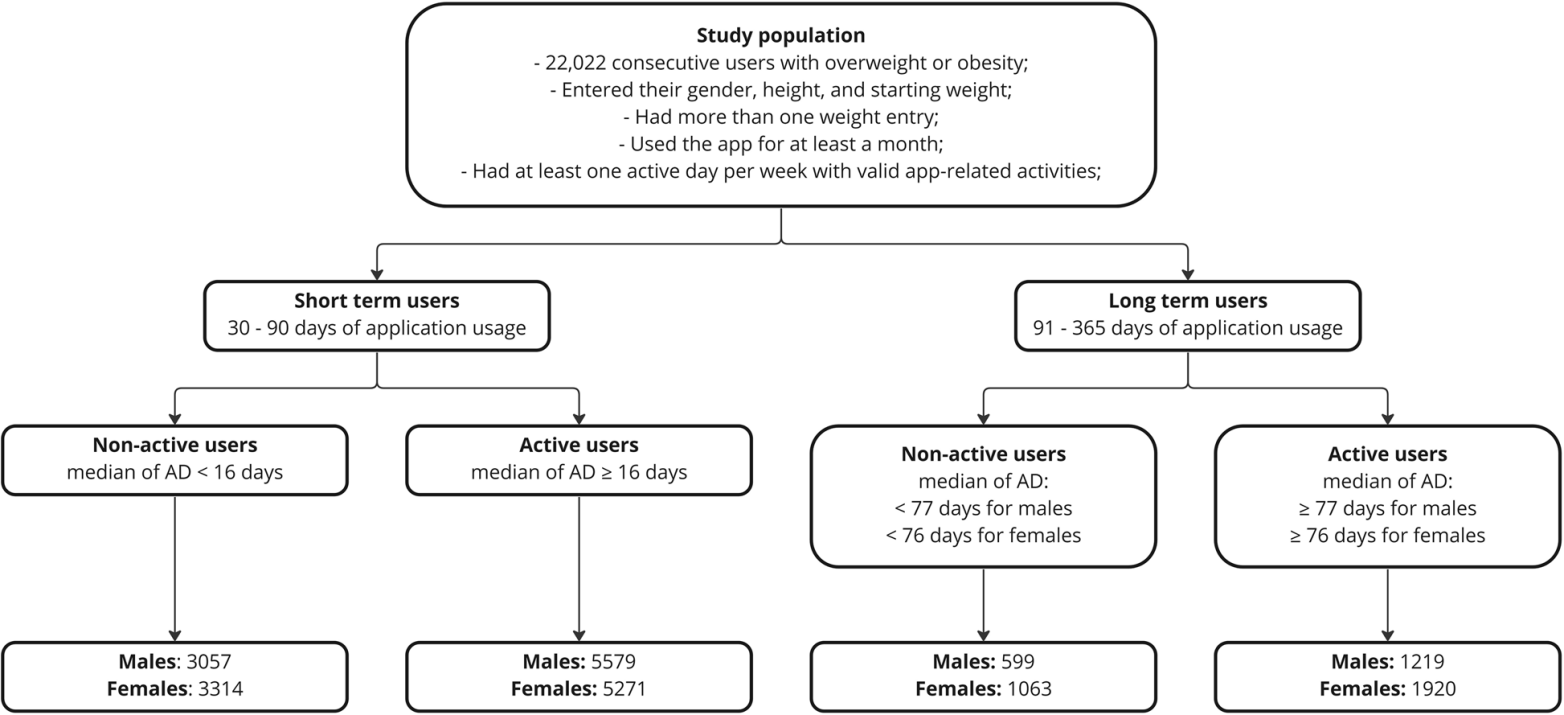
The flowchart of the study design and the number of patients who have been analyzed

The parameters retrieved from the database were self-reported and included gender, the number of active days (AD), total time of use (TT), starting and final weight, and height. During the preliminary data analysis, there was a significant user separation in usage time after approximately three months. A portion of users had dropped out by that time. Because of this empirical observation, those who used the application for less than 90 days were considered short-term users. Those who used the application longer were long-term users. The median of AD was used to divide the users into active and non-active groups. Starting and final BMI were calculated using the self-reported starting and final weight, respectively, according to the standard formula (weight (in kilograms) divided by squared height (in meters)). Three obesity classes were determined using BMI in accordance with CDC standards: ≥ 30 and < 35: Obese, Class I; ≥ 35 and < 40: Obese, Class II; and ≥ 40: Obese, Class III. Additionally, we did separate the male and female cohorts because of a major significant difference between the starting body weights (Fig. 1).

Firstly, the normalities of the data were checked by evaluating box and Q–Q plots, calculating the Z-scores of kurtosis and skewness, and using the Shapiro–Wilk test. The data with a normal distribution are presented using means and standard deviations (SD), and data with a non-normal distribution—using medians and interquartile ranges (IQR). The differences between the starting and final means of normally distributed data were compared using t-tests. The means between three or more groups were compared using one-way ANOVA. Prior to ANOVA, the data were checked using Levene ‘s test for equality of variances. If homogeneity of variances was not met, Welch ‘s ANOVA was used. If the data were not normally distributed, baseline and final means were compared using the Wilcoxon signed-rank test. The means of independent samples were compared using the Mann–Whitney U test. The difference between medians of the non-normally distributed data were compared using Mood ‘s median test. The chi-squared test of homogeneity was used to determine if a difference existed between the short- and long-term cohorts and between active and non-active cohorts. Post hoc analysis involved pairwise comparisons using the Z-test of the two proportions with a Bonferroni correction. The significance level for all tests was set at 5%. All statistical analyses were performed using IBM SPSS Statistics, version 26 (IBM Corp., Armonk, N.Y., USA).

## Results

The final study cohort comprised 22,022 users who were divided into short- and long-term cohorts, as described in the Methods section. The short-term cohort comprised 17,221 users (8585 females and 8636 males), while there were 4801 users (2983 females and 1818 males) in the long-term cohort. The distribution of users according to their starting BMI level in the short- and long-term cohorts can be seen in Table 1. The median active days of use for short-term users was 16 days, with an IQR of 20. It was the same for the male and female cohorts. The long-term user median of active days was 77 for males and 76 for females. Males tended to fast more than female users in the short- and long-term groups. The difference of medians of total fasting hours between male and female groups was statistically significant in both cohorts.

**Table 1:** Descriptive data regarding gender and usage time. User count is reported as numbers. Numbers of active days, fasts, and total fasting hours are reported as medians with an interquartile range in brackets

	**Short term (30–90 days)**		**Significance**	**Long term (91–365 days)**		**Significance**
	**Males**	**Females**		**Males**	**Females**	
Total number of users	8765 (36.6%)	9972 (41.6%)		1846 (7.7%)	3360 (14.0%)	
User count in starting weight groups:						
Overweight	2364 (31.7%)	3559 (47.8%)	***p* < 0.001**	434 (5.8%)	1090 (14.6%)	***p* < 0.001**
Obese, Class I	3770 (45.3%)	2776 (33.4%)		772 (9.3%)	1004 (12.1%)	
Obese, Class II	1841 (41.8%)	1534 (34.8%)		431 (9.8%)	599 (13.6%)	
Obese, Class III	661 (35.8%)	716 (38.7%)		181 (9.8%)	290 (15.7%)	
Active days of use	16 [20]	16 [20]	*p* = 0.144	77 [77]	76 [76]	*p* = 0.142
Fasts started	8 [13]	7 [11]	***p* < 0.001**	23 [49]	16 [39]	***p* < 0.001**
Total fasting hours	127 [202]	98 [162]	***p* < 0.001**	363 [757]	238 [612]	***p* < 0.001**

### Within-group weight loss analysis of users with overweight (active vs. non-active)

After dividing the users into non-active and active groups, the obtained weight change was compared in the cohort of users with overweight (Table 2). There was no statistically significant difference in starting weight between groups in the male and female cohorts. Long-term active and non-active male and female users had lost significant amounts of weight (Table 2). However, long-term active male and female users lost more weight than long-term non-active users did (males: 4.5 [5.8] vs 1.3 [4.3], *p* < 0.001; females: 4.0 [5.3] vs 1.1 [3.9], *p* < 0.001; the results are reported as medians with interquartile ranges). The same tendencies were found among short-term active and non-active males and females. Short-term active and non-active users lost significant amounts of weight (Table 2), and active users lost more weight than non-active users did (males: 1.8 [2.4] vs 1.4 [2.8], *p* < 0.001; females: 1.4 [2.1] vs 0.9 [2.1], *p* = 0.046).

**Table 2:** Starting and final body weights (kg) of users with overweight who were included in the study. The outcomes are distributed according to gender and activity level. The results are shown as medians with interquartile ranges in brackets

	**Short-term**			**Long-term**		
	**Non-active**	**Active**	**Sig**	**Non-active**	**Active**	**Sig**
**Males**						
Start	90.7 [10.2]	91.6 [11.3]	= 0.207	92.1 [12.3]	90.7 [10.3]	= 0.059
Final	89.6 [10.7]	89.1 [11.4]	= 0.556	90.7 [11.9]	85.6 [11.3]	** < 0.001**
Diff	1.4 [2.8]	1.8 [2.4]	** < 0.001**	1.3 [4.3]	4.5 [5.8]	** < 0.001**
Sig	** < 0.001**	** < 0.001**		** < 0.001**	** < 0.001**	
**Females**						
Start	74.4 [10.8]	74.0 [9.5]	= 0.631	74.0 [9.5]	74.8 [9.9]	= 0.354
Final	73.3 [10.5]	72.7 [9.6]	** = 0.020**	72.6 [10.4]	70.1 [11.0]	** < 0.001**
Diff	0.9 [2.1]	1.4 [2.1]	** < 0.046**	1.1 [3.9]	4.0 [5.3]	** < 0.001**
Sig	** < 0.001**	** < 0.001**		** < 0.001**	** < 0.001**	

### Between-group weight loss analysis of users with overweight (long term vs. short term)

Long-term users were then compared to short-term users (Table 2.). Active, long-term users lost more weight than active short-term users did (males: 4.5 [5.8] vs 1.8 [2.4], *p* < 0.001; females: 4.0 [5.3] vs 1.4 [2.1], *p* < 0.001). However, there was no statistically significant weight loss difference between non-active users in the short- and long-term cohorts among both genders.

### Within-group weight loss analysis of users with obesity (active vs. non-active)

Weight loss was compared among users with obesity as well (Table 3). There was no starting weight difference among active and non-active users in all groups. Additionally, users in all groups lost statistically significant amounts of weight, and active users tended to lose statistically significantly more weight than non-active users (Table 3).

**Table 3:** Starting and final body weights (kg) and their difference of users with obesity who were included in the study. The outcomes are distributed according to gender and activity level. The results are shown as medians with interquartile ranges in brackets

		**Short**			**Long**		
		**Non-active**	**Active**	**Sig**	**Non-active**	**Active**	**Sig**
**Males**							
Obese, Class I	Start	104.3 [12.3]	104.3 [12.3]	= 0.083	104.3 [12.3]	104.3 [10.9]	= 0.711
	Final	102.1 [12.2]	101.2 [12.1]	** < 0.001**	101.9 [13.1]	97.2 [13.6]	** < 0.001**
	Diff	1.8 [3.2]	2.3 [3.1]	** < 0.001**	1.6 [5.2]	5.9 [8.2]	** < 0.001**
	Sig	** < 0.001**	** < 0.001**		** < 0.001**	** < 0.001**	
Obese, Class II	Start	118.0 [13.9]	118.8 [12.9]	= 0.925	120.2 [13.6]	117.9 [14.5]	= 0.746
	Final	116.4 [13.8]	115.3 [14.3]	** = 0.032**	115.8 [13.5]	110.2 [16.2]	** < 0.001**
	Diff	1.9 [3.4]	2.7 [3.5]	** < 0.001**	1.8 [6.2]	6.7 [10.5]	** < 0.001**
	Sig	** < 0.001**	** < 0.001**		** < 0.001**	** < 0.001**	
Obese, Class III	Start	133.8 [15.9]	136.1 [17.8]	= 0.234	135.6 [14.5]	133.8 [14.1]	= 0.805
	Final	132.3 [18.1]	131.4 [18.3]	= 0.654	132.0 [22.1]	124.7 [18.5]	** = 0.034**
	Diff	2.5 [4.2]	3.4 [4.2]	** < 0.001**	2.6 [7.4]	8.0 [11.9]	** < 0.001**
	Sig	** < 0.001**	** < 0.001**		** < 0.001**	** < 0.001**	
**Females**							
Obese, Class I	Start	87.1 [10.9]	86.2 [10.9]	= 0.330	87.0 [11.4]	86.2 [10.5]	= 0.959
	Final	85.3 [11.2]	84.7 [11.5]	** = 0.009**	84.3 [12.3]	81.2 [12.5]	** < 0.001**
	Diff	1.2 [2.5]	1.6 [2.4]	** < 0.001**	1.9 [4.8]	4.9 [6.5]	** < 0.001**
	Sig	** < 0.001**	** < 0.001**		** < 0.001**	** < 0.001**	
Obese, Class II	Start	99.8 [11.8]	99.8 [11.8]	= 0.516	99.8 [10.1]	99.8 [13.6]	= 0.395
	Final	97.5 [12.7]	97.6 [12.2]	= 0.483	97.5 [12.1]	92.6 [14.6]	** < 0.001**
	Diff	1.4 [3.0]	1.9 [2.8]	** < 0.001**	2.4 [6.0]	6.5 [8.1]	** < 0.001**
	Sig	** < 0.001**	** < 0.001**		** < 0.001**	** < 0.001**	
Obese, Class III	Start	111.1 [11.4]	112.5 [15.0]	= 0.726	112.9 [13.6]	111.1 [12.7]	= 0.098
	Final	109.7 [13.8]	109.4 [14.5]	= 0.680	108.9 [13.0]	102.6 [13.5]	** < 0.001**
	Diff	1.6 [3.4]	1.8 [2.9]	** = 0.013**	3.7 [6.8]	7.8 [8.7]	** < 0.001**
	Sig	** < 0.001**	** < 0.001**		** < 0.001**	** < 0.001**	

### Between-group weight loss analysis of users with overweight (long term vs. short term)

When short-term users were compared to long-term users, the same tendencies were observed as in the overweight user group—active long-term users lost more weight than their counterparts in the active short-term group (males: 5.9 [8.2] vs 2.3 [3.1], *p* < 0.001, 6.7 [10.5] vs 2.7 [3.5], *p* < 0.001, 8.0 [11.9] vs 3.4 [4.2], *p* < 0.001; females: 4.9 [2.4] vs 1.6 [2.4], *p* < 0.001, 6.5 [8.1] vs 1.9 [2.8], *p* < 0.001, 7.8 [8.7] vs 1.8 [2.9], *p* < 0.001, for Obesity Classes I, II, and III, respectively). However, while weight loss did not differ between non-active males in the short- and long-term cohorts among all weight groups, non-active long-term female users lost significantly more weight than short-term non-active females did (1.9 [4.8] vs 1.2 [2.5], *p* < 0.001, 2.4 [6.0] vs 1.4 [3.0], *p* = 0.014, 3.7 [6.8] vs 1.6 [3.4], *p* < 0.001, for Obesity Classes I, II, and III, respectively).

### Changes of BMI

Furthermore, users were stratified according to their initial BMI level to analyze how many users reduced their BMI level while using the application. It was found out that 2842 users (15.3%) in the short-term group lowered their BMI level, compared to 1666 users (32.8%) in the long-term group. There was a statistically significant difference between the proportions (*p* < 0.001). The same tendency was observed with non-active vs active users. There was a statistically significant difference in the proportion between non-active and active cohorts in terms of BMI level reduction (14.7% vs 21.6%, *p* < 0.001). Moreover, 344 users managed to reduce their initial BMI level from obese or overweight to normal.

### Multiple regression analysis

Finally, a multiple regression model was created to find which reported activities and other covariates contributed the most to weight loss (Table 4). The multiple regression model statistically significantly predicted weight loss, F(11, 23,931) = 739.695, *p* = 0.000, R^2^ = 0.236, adj. R^2^ = 0.236. Only guide reading, step tracking, and starting a workout did not add statistical significance weight loss prediction. Regression coefficients and standard errors can be found in Table 4. As can be seen from the regression coefficient column, being an active long-term user meant statistically significant weight loss that was more than half a kilogram. One extra hour of fasting gave approximately three grams of lost weight. Weight tracking and completing daily challenges further statistically significantly lowered bodyweight by an additional 25 and 20 g, respectively.

**Table 4:** Multiple regression results for weight loss

Weight loss	B	95% CI for B		Sig	SE B	R^2^	ΔR^2^
		LL	UL				
Model						23.6%	23.6%
Constant	0.743	0.587	0.898	**0.000**	0.079		
Total time of usage	0.273	0.147	0.399	**0.000**	0.064		
Number of active days	0.340	0.249	0.432	**0.000**	0.047		
Total Fasting Hours	0.003	0.003	0.003	**0.000**	0.000		
Weight Tracking	0.025	0.023	0.027	**0.000**	0.001		
Completing Challenges	0.020	0.017	0.022	**0.000**	0.001		
Guide Reading	0.016	0.007	0.025	0.062	0.005		
Step Tracking	0.026	0.005	0.047	0.059	0.011		
Weekly Summary Screen Visited	0.023	0.016	0.030	**0.000**	0.003		
Fasting Feeling Feedback Given	0.004	0.001	0.007	**0.015**	0.002		
Workout Started	–0.006	–0.025	0.012	0.500	0.010		

Model: „Enter “ method in SPSS; B, regression coefficient; *CI* Confidence interval, *LL* Lower limit, *UL* Upper limit, *SE B* Standard error of the coefficient, *R*^*2*^ Coefficient of determination, *ΔR*^*2*^ Adjusted R^2^

## Discussion

Our primary finding in this retrospective study is that active long-term DoFasting users statistically significantly reduced their bodyweight. Moreover, active long-term users statistically significantly lost more weight than non-active users and short-term active users did. Generally, the trend was that the higher the starting weight, the more weight was lost. Additionally, long-term and active users were more likely to reduce their BMI level.

It has been demonstrated in previous research that IF results in weight loss. In a recent systematic review, Welton et al. [19] conclude that all included IF trials showed weight loss (0.8–13.0%) without adverse events and thus, IF shows promise for the treatment of obesity. Another systematic review by Patterson et al. also found that almost any IF regimen results in weight loss, and in most trials, statistically significant weight loss is observed [9]. Unfortunately, research on the effectiveness of IF guided with a mobile application is scarce. Significant weight loss has been demonstrated with other dietary interventions delivered through an app, such as a ketogenic diet (which shares some mechanisms with fasting) [20], but not with IF. In light of this, our study of the effectiveness of IF facilitated through a mobile app is relatively novel. Our results not only add to the knowledge about weight loss with IF but also provide insight into whether and how effectively IF can be facilitated by a mobile app.

It has been shown in mHealth studies that engagement with the apps is an important predictor for weight loss [21, 22]. A large study that reported successful weight loss while using a mobile app also found that users who actively logged their exercises were more likely to lose weight [23]. Our comparisons of the long-term active group to the short-term active group and the long-term non-active group also illustrate this very important point: the best results can be expected when users adhere to the app for longer and, importantly, engage with it. Using the app inactively, even for a longer time, would likely not give the desired outcomes. As can be seen from the results, even short-term active users tended to lose at least as much weight as long-term non-active users. Therefore, engagement with the app is of paramount importance for the effectiveness of the intervention, and long-term adherence could be expected to sustain and improve the results even further.

To investigate engagement further, we created a multiple regression model to see which activities predicted weight loss. Length of use and in-app activity were the strongest statistically significant predictors. Most app activities statistically significantly contributed to the model, albeit with varying contributions. Of particular interest is the finding that step tracking and starting workouts were not significant predictors for weight loss. Physical activity is proven to be a pivotal part in weight loss and its maintenance [24]. However, there are substantial data indicating that weight loss with IF regimens is mostly fat loss, while more lean mass is preserved [19, 25]. Thus, it could be that users who were more engaged with exercises, combined with IF, lost mostly fat but preserved or gained more muscle mass, therefore losing less weight. A more established framework for evaluating engagement metrics is needed in mHealth research. Currently, app feature inclusion stems mostly from theoretical mHealth app design, and evaluating which features contribute the most and how is difficult. To add the most to the knowledge base, future studies in this field should attempt to analyze engagement variables.

There are certain promising benefits that mobile apps bring to diet interventions. Some researchers argue that apps are able to facilitate diet-related behavioral change by providing relevant guiding material to their users and improving motivation and setting of goals [18]. It was concluded by King et al. that smartphones can be platforms for effective delivery of health interventions [26]. App effectiveness has also been demonstrated in trials. A pilot controlled trial has demonstrated that smartphone apps are superior to conventional intervention delivery in terms of adherence [17]. In a recent systematic review, it is argued that particular features of mHealth apps, such as guiding materials, reminders and feedback, setting of goals, and visualization facilitate the management of overweight-related conditions [27]. Users of the mobile application that was used in this investigation are provided with some of the functionalities that are mentioned as effective in the literature. The app tracks and gives appropriate timed prompts regarding fasting cycles, which acts as a reminder. An articles section within the app gives users diet-related educational material. The users are also encouraged to track their progress via logging and are able to set goals and visualize their progress in the app. The possibility to take up fasting challenges within the app is of particular interest. Challenging oneself and receiving appropriate feedback can improve self-management through the app by „gamifying “ and bringing a specific appeal to the whole process, some researchers argue [28]. The gamification of health apps is a trending field that will likely receive increasing attention in the future. An in-depth conceptual gamified model for IF app design has been proposed, hoping to increase app effectiveness and retention rate [29].

Some strengths and limitations of the present study are worth noting. The retrospective analysis allowed us to access a relatively large user sample, which improved the robustness of our results. Creating a predictive model for app features gave insights into which app design decisions may be worth investigating. However, the current study was not prospective and thus did not have randomization or a control group. On top of that, the users’ results were self-reported, which was subject to bias. Therefore, we cannot at the moment provide strong conclusions that our results were given entirely by the use of the mobile application only. Weight loss is a complex matter that involves covariates such as comorbidities and habits of diet and physical activity. Furthermore, as mentioned earlier, IF targets mainly fat loss, but we were unable to track the users’ body composition in the present study. Thus, it is difficult to compare our results to those of other IF studies in terms of body composition changes. A longer study period for future studies would also be desirable to estimate how the obtained weight changes are sustained. It has been observed that behavioral interventions with mobile health apps give significant results, but they have a tendency to diminish over time [30]. Finally, as discussed above, defining and evaluating engagement in mHealth apps is relatively novel, and analyses such as ours require further conceptual investigation and validation. We are planning a prospective controlled trial in the future to study the effectiveness of DoFasting and other mobile applications that are available to improve upon some of these limitations and provide more robust results.

## Conclusions

Body weight and in-app activity metrics of DoFasting users were analyzed in current study. We found that long-term active users of the app lost a statistically significant amount of weight over the study period and lost more than inactive users did. Active and long-term users were also more likely to reduce their BMI class. Furthermore, we created a multiple regression model to investigate which features were predictive of weight loss. Total length of use and active days were the most significant predictors of weight loss. In-app activities such as total fasting hours, weight logging, completing challenges, and providing feedback were also statistically significant predictors of weight loss with varying contributions. A follow-up study will be done to analyze how weight loss relates to changes in body composition while using the DoFasting mobile application.

## Data Availability

The datasets generated and analyzed during the current study are not publicly available due to protection of confidential information of Kilo Health business and Kilo Health users but are available from the corresponding author upon reasonable request.

## Abbreviations

IF: Intermittent fasting
ADF: Alternate-day fasting
ADMF: Alternate-day modified fasting
TRE: Time-restricted eating
CR: Caloric restriction
